# Novel Splice Site Pathogenic Variant of *EFTUD2* Is Associated with Mandibulofacial Dysostosis with Microcephaly and Extracranial Symptoms in Korea

**DOI:** 10.3390/diagnostics10050296

**Published:** 2020-05-12

**Authors:** So Young Kim, Da-hye Lee, Jin Hee Han, Byung Yoon Choi

**Affiliations:** 1 Department of Otorhinolaryngology-Head and Neck Surgery, CHA Bundang Medical Center, CHA University, Seongnam 13496, Korea; sossi81@hanmail.net (S.Y.K.); ldada72@naver.com (D.-h.L.); 2 Department of Otorhinolaryngology-Head and Neck Surgery, Seoul National University Bundang Hospital, Seongnam 13496, Korea; flyswan@hanmail.net

**Keywords:** *EFTUD2*, mandibulofacial dysostosis, exome sequencing, splicing

## Abstract

Elongation factor Tu guanosine-5’-triphosphate (GTP) binding domain containing 2 (*EFTUD2*) encodes a major component of the spliceosomal GTPase and, if mutated, causes mandibulofacial dysostosis with microcephaly (MFDM; MIM#610536). Despite the increasing number of potentially pathogenic variants reported in the literature, most previous studies have relied solely on in silico prediction of the pathogenic potential of *EFTUD2* variants, which may result in misclassification of the variant’s pathogenicity. Given the importance of the functional verification of *EFTUD2* variants, we identified a novel splice donor site variant, c.271+1G>A of *EFTUD2*, whose pathogenicity was clearly verified at the RNA level using a minigene assay. A child with MFDM, mixed hearing loss, microcephaly, and a congenital cardiac defect was identified with this variant, which arose in a de novo fashion. The minigene assay showed erroneous integration of the 118 bp IVS3 of *EFTUD2* exclusively among the c.271+1G>A variant clone. We first applied the minigene assay to identify the splice function of a splice site variant of *EFTUD2*, thereby allowing for in vitro functional verification of splice site variants in *EFTUD2*.

## 1. Introduction

Mandibulofacial dysostosis with microcephaly (MFDM) or mandibulofacial dysostosis type Guion–Almeida (MIM#610536) is a craniofacial malformation syndrome, primarily in the first and second branchial arches [1,2]. The causative gene for MFDM, if mutated, is elongation factor Tu GTP binding domain containing 2 (*EFTUD2*), which encodes the U5 spliceosomal GTPase. A growing number of pathogenic variants of *EFTUD2* have been identified since 2012 [2,3,4,5,6]. An *EFTUD2* variant is inherited in an autosomal-dominant pattern, and about 75% of the variants demonstrate de novo inheritance [3]. The clinical presentations of MFDM are composed of classic craniofacial features with MFDM, malar hypoplasia, zygomatic cleft, external and/or inner ear anomalies, choanal atresia, and microcephaly [2,5]. The severity of these features is variable among affected probands. In addition, there are numerous minor features, including central nervous system disorders, esophageal atresia, congenital cardiac defect, and genitourinary diseases [5,7,8].

The diagnosis of MFDM has often required multiple steps of molecular genetic studies because it is rare and has a wide spectrum of phenotypic manifestations. The phenotypes overlap with other craniofacial disorders, including oculo-auriculo-vertebral spectrum (OVAS), CHARGE, Pierre–Robin, and Treacher Collins syndrome, which warrants the differential molecular diagnosis of these genetic diseases [9,10]. Indeed, a considerable number of MFDM patients are evaluated using chromosomal microarray, targeted gene sequencing of *CDH7*, *FGFR2***,** and other pathogenic genes for craniofacial anomalies, along with examinations of copy number variation [10]. Exome sequencing (ES) has been used to identify pathogenic variants of *EFTUD2* in patients who remain undiagnosed after molecular genetic studies [10,11,12]. However, the damaging effects of the *EFTUD2* variant have been verified in only a small number of cases [3]. Indeed, many studies have predicted pathogenic effects based solely on in silico analyses. In addition, although the pathogenic variants and application of next-generation sequencing have improved the genetic diagnosis of MFDM, its broad and heterogeneous phenotypic spectrum and numerous variants of uncertain significance complicates the molecular etiologic diagnosis and supports the importance of documenting the pathogenic effects of *EFTUD2* variants.

We recently encountered a singleton with MFDM and mixed hearing loss, who was found to carry a novel, de novo splice donor site variant of *EFTUD2* through ES. The pathogenic effects of the variant on the canonical splice donor site were demonstrated using a rigorous functional analysis.

## 2. Materials and Methods

### 2.1. Patients

The Ethics Committee of Bundang CHA Medical Center (2018–06–008–014) approved this study. Written informed consent was obtained from the parents of the patients. All study protocols complied with regulations of the institutional ethical committee of Bundang CHA Medical Center. A child who failed in the newborn hearing screening tests was referred for evaluation.

### 2.2. Clinical and Audiologic Evaluation

Physical examination and systemic review were performed by pediatricians. Imaging studies were conducted, including brain MRI and echocardiogram. Family history was obtained from the parents. A tympanic endoscopic examination was performed to evaluate the tympanic membrane by an otologic doctor. Audiologic evaluations were performed using distortion product otoacoustic emission (DPOAE), auditory brainstem response (ABR), and auditory steady state evoked response (ASSR).

### 2.3. Chromosomal and Targeted Gene Studies

Chromosomal study was performed to exclude cytogenetically visible chromosomal abnormalities. To exclude Cornelia de Lange syndrome (OMIM 122470), direct sequencing of *NIPBL* coding exons and their flanking introns was performed.

### 2.4. Exome Sequencing, Variant Calling, and Variant Annotation

Genomic DNA was extracted from buccal swab samples of the proband. Whole exons and their flanking intronic regions were captured using Agilent SureSelect kits (version C2, December 2018) and sequenced using the NovaSeq platform (Illumina, San Diego, CA, USA). Raw genome sequencing data were aligned to the human reference sequence GRCh37 (hg19). Differences were called and annotated. The mean depth of coverage was 100X (>10X = 99.2%). The variant interpretation was performed as described previously [13,14]. Variants with minor allele frequency (MAF) < 0.005 were selected based on the population genomic databases of ExAC (http://exac.broadinstitute.org/), 1000 Genomes (https://www.ncbi.nlm.nih.gov/variation/tools/1000genome), GO-ESP (http://evs.gs.washington.edu/EVS/), GnomAD (http://gnomad.broadinstitute.org/), and KRGDB consisting of 1722 Korean individuals (3444 alleles; http://coda.nih.go.kr/coda/KRGDB/index.jsp). Splice site variants were analyzed on the basis of in silico splice predictors, such as MaxEntScan [15]. The prioritized variants were categorized based on the 2015 American College of Medical Genetics and Genomics-Association for Molecular Pathology (ACMG-AMP) guideline for the interpretation of sequence variants [16]. ExPASy was used to predict the translation product of a variant (https://web.expasy.org/translate/).

### 2.5. Validation of the EFTUD2 Variant and TRIO Studies

Validation of the *EFTUD2* splice variant of c.271+1G>A was performed using Sanger sequencing from genomic DNA from the proband and his parents using primers (F:5′-CATATCAGGCCACAGGGTAA-3’, R:5′-CTCCCTCTCCCTCTTGCTTT-3′).

### 2.6. Minigene Assay

To explore the effects of the splice site variant (c.271+1G>A) on intron 3, an in vitro splicing assay was performed using the minigene assay. Wild-type and mutant *EFTUD2* exon 3 (166 bp) and its flanking intron 2 (297 bp) and intron 3 (288 bp) were amplified using primers containing additional EcoRl and Ndel restriction sites. The amplified fragments and pSPL3 exon trapping vectors were digested with a restriction enzyme. Exon A and exon B were cloned into pSPL3 vectors. The wild type and mutant-containing vector sequences were confirmed with Sanger sequencing.

Approximately 3 × 10^5^ COS-7 cells (monkey kidney fibroblast-like cell line) were plated in four-well plates (Nunc, Roskilde, Denmark). They were grown to 90% confluency in 0.5 mL medium (DMEM, 10% fetal bovine serum, 2 mM glutamine, 1% non-essential amino acids and 1% penicillin/streptomycin). Then, 5 µg minigene was transfected into the COS-7 cells using low toxicity Lipofectamine 3000 Reagent (Thermo Fisher Scientific, Waltham, MA, USA) in Gibco^TM^ Opti-Mem^TM^ medium (Thermo Fisher Scientific, Waltham, MA, USA). Cells were incubated for 48 h. RNA was extracted using the Rneasy Mini Kit (Qiagen, Hilden, Germany).

Total cellular RNA was extracted from the sample-treated cells using the Rneasy Mini Kit (Qiagen, Hilden, Germany). Total RNA (5 μg) was converted into single-stranded cDNA using a KOD-Plus-Neo (TOYOBO, Osaka, Japan). This cDNA was amplified using the pSPL3 vector-specific primers SD6 (5′-TCTGAGTCACCTGGAC AACC-3′) and SA2 (5′-ATCTCAGTGGTATTTGTGAGC-3′). The amplification cycles were 94 °C for 30 s, 60 °C for 30 s, and 72 °C for 40 s. After 40 cycles, PCR products were separated by electrophoresis on 2% agarose gels for 30 min at 100 V. Then, the gels were stained with RedSafe (Intron, KOR).

## 3. Results

### 3.1. Clinical Manifestations

**Figure 1:**
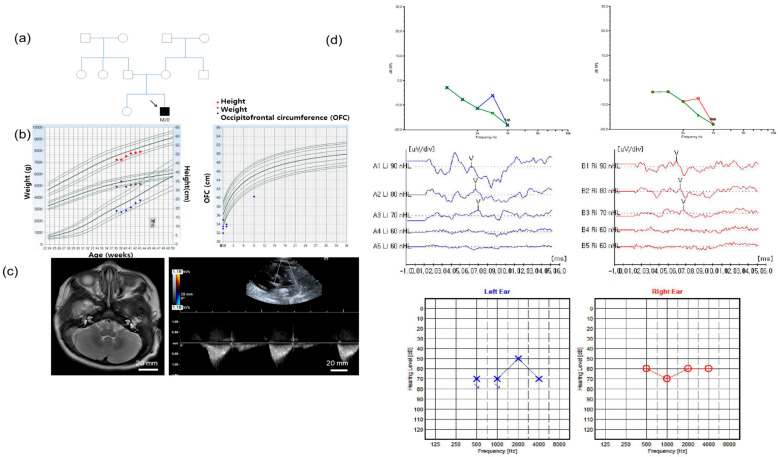
Auditory evaluations, imaging studies, and pedigree of the proband. (**a**) The proband was the only affected individual in the family; (**b**) The growth parameter of weight, height, and occipitofrontal circumference of the proband; (**c**) Brain MRI showed normal inner ear morphology and no evidence of other brain anomalies. Echocardiogram showed abnormal and echogenic density in the pulmonary artery area and tricuspid valve insufficiency; (**d**) Distortion product otoacoustic emission (DPOAE), auditory brainstem response (ABR) thresholds, and auditory steady state response (ASSR) tests demonstrated hearing loss of the proband (red line, o = right ear, blue line, x = left ear).

A 3-month-old male patient was referred to the otology clinic due to failure in the newborn hearing screening test. He is the second baby of healthy nonconsanguineous parents (Figure 1a). He was born at 38 + 0 weeks gestational age via normal spontaneous vaginal delivery without any perinatal events. The Apgar score was 8 points at birth. His birth weight was 2.945 kg (<25 centile), and occipitofrontal circumference (OFC) was 30.5 cm (<1 centile) (Figure 1b). He showed facial dysmorphology with microcephaly (<3 centile at 9 months old), mild low set, microtia, and micrognathia. Psychomotor, somatic, and speech development were delayed. Brain magnetic resonance imaging (MRI) was performed at the age of 3 months, and there were no pathological findings such as ischemia, hemorrhage, mass, atrophy, or hydrocephalus in the cerebrum, cerebellum, brainstem, ventricle, or cerebrospinal fluid space (Figure 1c). The inner ear also had normal morphology. The echocardiogram, which was performed at the age of 3 months, showed abnormal echogenicity in the pulmonary artery area and tricuspid valve insufficiency.

The distortion product otoacoustic emission (DPOAE) response, which was performed at the age of 3 months, was absent. The average auditory steady state evoked response (ASSR) thresholds, which was performed at the age of 4 months, were 65 dB Hearing Level (HL) in the right ear and 60 dB HL in the left ear (Figure 1d). The auditory brainstem response (ABR) thresholds, which were performed at the age of 4 months, were 70 dB Sound Pressure Level (SPL) in both ears. The tympanic membranes were filled with serous effusions on both sides of the ears. Ventilation tubes were inserted in both ears at the age of 10 months. After surgery, the repeated ABR thresholds, which were performed at the age of 12 months, were checked as 30 dB SPL in the right ear and 20 dB SPL in the left ear. There was no family history of hearing loss or other syndromic disorders, including mandibulofacial anomalies.

### 3.2. Differential Molecular Genetic Diagnosis

**Figure 2:**
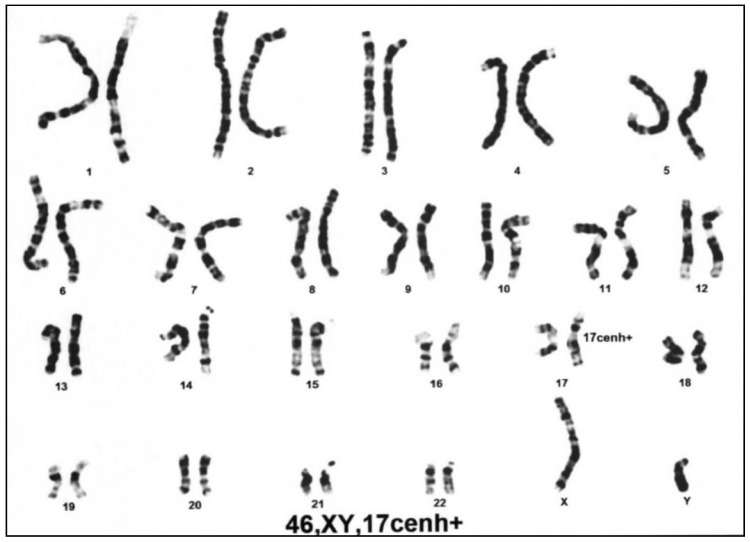
The cytogenetic chromosomal study showed no abnormal finding in the 46 XY chromosomes, excluding the normal variation in 17 centromeric heterochromatin.

Due to the dysmorphology of low set ears, micrognathia, and microcephaly, chromosomal studies were performed to identify chromosomal anomalies, including duplication 3q syndrome at the age of 1 week. There were no abnormal findings in the 46 XY chromosomes, excluding the normal variation in 17 centromeric heterochromatin (Figure 2).

Direct sequencing of the 46 coding exons of the Nippled-B-like protein (*NIPBL*) gene was performed at the age of 2 weeks to exclude Cornelia de Lange syndrome (OMIM 122470), revealing no variants that could be categorized as pathogenic, likely pathogenic, or uncertain significance (VUS) according to the 2015 ACMG and 2018 ACMG guidelines [16,17].

### 3.3. Identification of a Novel Splice Site EFTUD2 Variant

ES, which was performed at the age of 8 weeks, and following variant prioritization based on the phenotypic features of hearing loss, dysmorphology, and developmental delay, revealed a splice donor site variant of *EFTUD2* (c.271+1G>A). No other second-tier candidate variant was selected. The c.271+1G>A was a novel splice donor site variant that was not detected in the populational database, including the Korean Reference Genome Database (KRGDB). Using in silico analyses, the Exon Splicing Enhancer Finder (ESE) finder result was −3.70, and MaxEntScan result was −0.39, which was predicted to disrupt the splice site of IVS 3.

**Figure 3:**
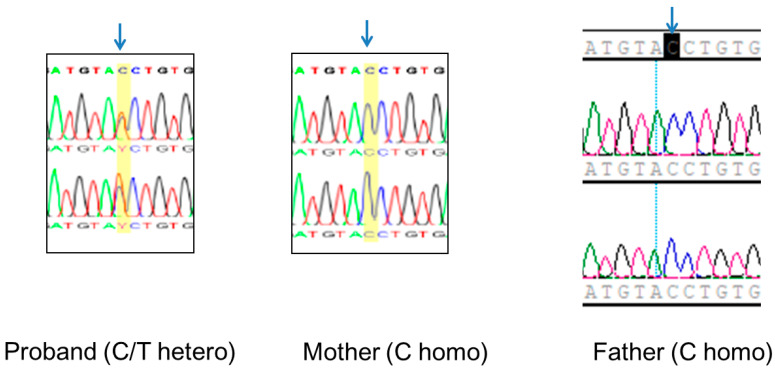
The Sanger sequencing chromatograms of the proband–parents TRIO study for *EFTUD2* c.271+1G>A variant showed wild type (C homo) in both parents and single heterozygote (C/T hetero) in the proband. (blue arrow = variant position)

Sanger sequencing of the proband confirmed the c.271+1G>A of *EFTUD2* as a single heterozygous variant. Sanger sequencing on the proband and his parents (“TRIO” testing) confirmed the de novo occurrence of c.271+1G>A of *EFTUD2* in the proband (Figure 3).

### 3.4. In Vitro Functional Characterization of c.271+1G>A

**Figure 4:**
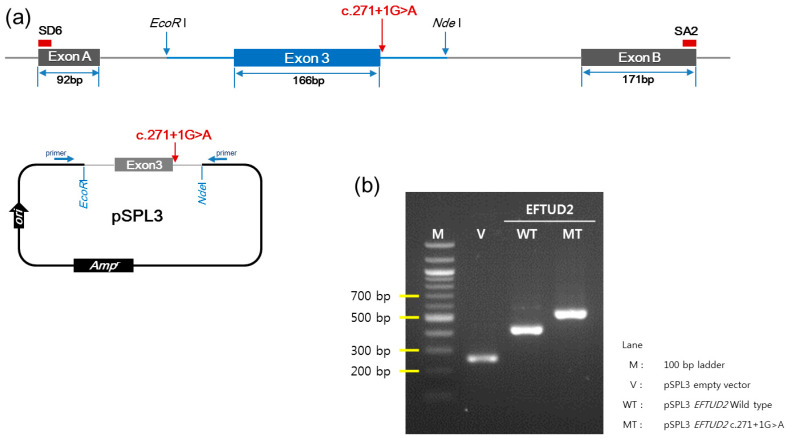
The minigene assay for a novel splice site variant of *EFTUD2* c.271+1G>A. (**a**) The design of the pSPL3 exon trapping vectors with exon A and B, which flanked exon 3 with the intronic sequence of *EFTUD2*; (**b**) PCR amplification of wild-type and c.271+1G>A variant cDNA revealed an abnormally large transcript in the c.271+1G>A variant clone (547 bp) compared to wild-type clone (429 bp).

**Figure 5:**
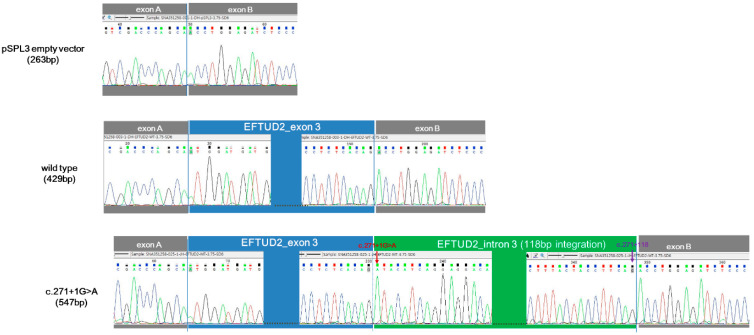
The Sanger sequencing of cDNA revealed aberrant integration of 118 bp intron 3 of *EFTUD2* between exon 3 and exon B in the c.271+1G>A variant clone. The wild type (429 bp) and empty vector (263 bp) yielded sequences as predicted.

**Figure 6:**
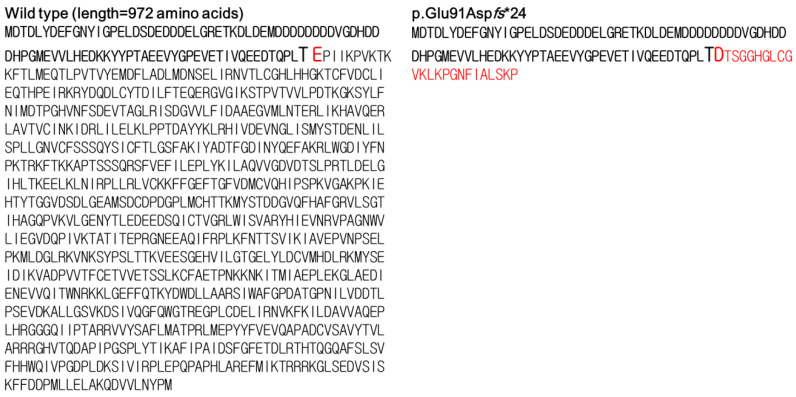
In silico prediction of the effect of the c.271+1G>A variant on translation, compared to the wild-type. The full protein (**left**) was expected to be truncated (**right**) in the c.271+1G>A variant. The red letters were indicated the amino acid residues that were altered due to the aberrantly spliced product.

The minigene assay was conducted for c.271+1G>A. PCR amplification of wild-type and mutant cDNA revealed a different sized product (Figure 4). The c.271+1G>A variant clone resulted in an approximately 500-bp cDNA sequence, which was larger than the 429 bp wild-type clone. Sanger sequencing of cDNA revealed a 547-bp sequence due to the aberrant integration of 118-bp IVS3 of *EFTUD2* between exon 3 and exon B exclusively in the c.271+1G>A variant clone (Figure 5). The wild type (429 bp) and empty vector (263 bp) yielded sequences as predicted. The translation product of c.271+1G>A variant was expected to be truncated by 11.7% (114/972 amino acids) in silico prediction using the Expert Protein Analysis System (ExPASy; https://web.expasy.org/translate/; Figure 6).

Finally, according to the ACMG 2015 and 2018 guidelines [16,17,18], the c.271+1G>A variant was classified as pathogenic (PVS1, PS2, and PM2). The c.271+1G>A variant has been submitted to LOVD v3.0 under accession ID #00289358 (http://databases.lovd.nl/shared/individuals/00289358).

## 4. Discussion

*EFTUD2* has been reported to be a pathogenic gene that causes MFDM with a wide phenotypic spectrum. In this study, we identified a novel, de novo splice donor site variant of c.271+1G>A in a Korean proband who manifested the classic triad of malar hypoplasia, micrognathia, and mandibular hypoplasia, in addition to mild low-set microtia, mixed hearing loss, and a congenital cardiac defect of tricuspid insufficiency. Although this proband had classic phenotypes of MFDM, it could be diagnosed only after multiple molecular genetic studies due to the rare occurrence of this disease and the broad phenotypic spectrum. A chromosomal study, targeted gene sequencing, and ES were used to diagnose MFDM. He was initially subjected to chromosomal study and targeted gene analyses, all of which failed to establish the genetic diagnosis. To validate the effects of splice donor site variants, PCR amplification and Sanger sequencing of the cDNA product of the splice donor site variant were performed. The splice donor site variant skipped the normal splicing site, and an abnormally large transcript was detected.

The c.271+1G>A variant is a novel, de novo splice donor site variant that disrupts the canonical splice donor site of IVS3. Integration of a 118-bp intronic sequence, as predicted using the minigene assay, would result in early termination of translation or dysfunction of the GTP-binding domain of a variant transcript. At this time, a total of 119 *EFTUD2* variants, including 16 pathogenic or likely pathogenic variants, 10 VUS, 14 benign or likely benign variants, and 79 unclassified variants, have been reported in the *EFTUD2* mutation database, which was last updated on 4 December 2019 (http://databases.lovd.nl/shared/genes/EFTUD2). About 82% of the *EFTUD2* variants have been classified as truncation variants, such as stop-gain and splice site variants [3]. There was no definite mutational hot spot in the *EFTUD2* gene, and the identified pathogenic *EFTUD2* variants were distributed throughout the exons [3].

The pathogenic potential of many splice site variants of *EFTUD2* was predicted using only in silico analyses, thus lacking functional experimental studies. A total of 37 splice variants have been reported in *EFTUD2*, including the current case [2,3,5,8,10,12,19,20,21,22,23] (Table S1). The aberrant transcript product was verified only for four splice site variants and the current variant at the RNA level [3]. The four splice site variants were c.1859A>T; 1860 + 3_1860 + 4delinsGAG (p.Ala574_Lys620del), c.1963–2A>T (p.Val655Glufs*8), c.105G>A (p.Thr3_Asp37del), and c.272–11_280del (p.Glu91Valfs*6). To test these variants, RT-PCR was performed with RNA from either lymphoblast or fibroblast as a template [3]. However, the splicing products of the cochlea could not be evaluated in this study. Among these four splice site variants, c.1859A>T; 1860 + 3_1860 + 4delinsGAG (p.Ala574_Lys620del) was initially reported as c.1859A>T (p.Lys620Met) in subjects with MFDM with esophageal atresia and choanal atresia in Polish populations [8]. The splice site variant (1860 + 3_1860 + 4delinsGAG) was identified only after RT–PCR on either lymphoblast or fibroblast RNA [3]. The remaining 32 splice site variants await functional verification.

The splice site variants could be misclassified as pathogenic when interpreted without functional studies. A heterozygous 5-bp deletion in IVS 12 (c.1058 + 3_1058 + 7del) of *EFTUD2* was initially reported as a causal variant in a subject with craniofacial anomaly, esophageal atresia, congenital cardiac defect, and microtia [23]. However, a minor allele frequency in gnomAD was estimated to be 1571/282,140 (5.568%) with 6 homozygotes and is now presumed to be a non-pathogenic variant (https://gnomad.broadinstitute.org/). RNA RT–PCR analysis of another gene with a pathogenic mechanism of haploinsufficiency, myosin-binding protein C (MYBPC3), reclassified the splice site variants from VUS to likely pathogenic variant in 7% (4/56) families [24]. However, the splicing errors in two likely pathogenic variants of MYBPC3 of c.927-9G>A and c.3190 + 5G>A, denoted by minigene assay, could not be recognized by RNA PCR of peripheral blood [24]. Minigene assay, combined with computational rare variant prioritization, could identify 48 pathogenic splicing variants of two autosomal dominant haploinsufficiency genes of lamin a/c (*LMNA*) and *MYBPC3*, which were previously classified as uncertain significance [25]. In addition, not all canonical splice site variants were completely abolished at the splice site, and sometimes normal splice site products can be generated from the canonical splice site variant, leading to a milder phenotype [26]. Affected individuals with canonical acceptor splice site variant c.652-2A>C in the *COL11A1* gene demonstrated mild to moderate degree of sensorineural hearing loss [26]. Minigene assay of this variant showed both aberrant splicing transcript skipping exon 5 and normal transcript, and this implied the leaky splicing function [26]. In addition, the effects of splice site variants could be differentiated based on cryptic splice sites, exon skipping, and partial or complete retention of introns [18,27]. Although the 2015 ACMG guidelines designated the canonical splice site variants in a gene with a loss-of-function mechanism as a very strong level of pathogenicity (PSV1), they recommended conducting RNA studies considering the effects of splice site variants [18]. Moreover, the splicing function could vary according to the target organ. Thus, functional documentation of the pathogenic effects of this splice site variant is important.

The individual with c.271+1G>A variant in *EFTUD2* of the current study showed phenotypic features of mixed hearing loss and congenital cardiac defect, as well as MFDM. Individuals with MFDM have been reported to present cardinal phenotypes of microcephaly and mandibulofacial anomalies in most cases and minor phenotypes, including hearing loss and congenital cardiac defects. Sensorineural hearing loss and cardiac defects are less common phenotypic features estimated to be present in less than 30% of the individuals with MFDM [3,20,21]. Among the individuals with a splice variant of *EFTUD2*, 14 of 37 individuals (37.8%) were reported to have SNHL or mixed hearing loss (Table S1). The congenital cardiac defect has been reported in 8 of 37 (21.62%) individuals with splice site variants. These proportions were comparable with those in all individuals with MFDM. The phenotype–genotype correlation has been ill-defined for MFDM. Yu et al. explored the phenotype potentially related to the splice site variants of *EFTUD2*. Specifically, they reported that ossicular abnormalities were significantly less in cases with splice site variants than those with non-splice site variants (33.3% [3/9] in splice site variants vs. 88.9% [8/9] in non-splice site variants, *p* < 0.05) [21]. However, the number of cases reported for the presence of ossicular anomaly was too small, only in 9 individuals with splice site variants, compared to the total number of reported cases of MFDM [21], which limited the reliability of the results.

Defective mRNA maturation due to haploinsufficiency of the spliceosomal function is believed to cause MFDM [23]. U5–116 kD, which is encoded by *EFTUD2*, consists of a GTP-binding domain and highly conserved domains II–V and is believed to regulate splicing function and disassembly of the spliceosome through the “GTP switch” [28,29]. Although the pathogenic signaling pathway of *EFTUD2* is not fully defined, previous studies have identified candidate signaling pathways relevant to *EFTUD2* variants [30,31]. The single heterogeneous *EFTUD2* mutant zebrafish model, which was identified by positional cloning of an ethylnitrosourea-induced zebrafish mutants, *fn10a*, and induced by *eftud2^fn10a^* morpholino injections, showed brain malformation and neuronal apoptosis via activation of the p53 pathway [30]. Similarly, with human osteoblast and chondrocyte cell lines with knockdown of *EFTUD2* using scrambled shRNAs, RNA-Seq analyses characterized the activation of p53 signaling pathway genes [31]. Additionally, because the expression of *EFTUD2* is not restricted to the first and second pharyngeal arches and is widely expressed in the mandibular mesenchyme, mesenchyme of the limb and lung, trachea, esophagus, and epithelium of otic vesicles, the pathogenic *EFTUD2* variants could have broad phenotypic presentations [23].

Although our results confirmed the abolished splice site and erroneous insertion of IVS3 in a splice variant in vitro, the in vivo functional effect remains unclear. Due to the fact that the reported cases of MFDM are rare (approximately 130 cases), the *EFTUD2* variant interpretation for pathogenic potential using population data is limited. The present study improves the pathogenic *EFTUD2* variant interpretation using the minigene assay. The aberrant splice product was also identified using the minigene assay.

## Supplementary Materials

Supplementary materials can be found at https://www.mdpi.com/2075-4418/10/5/296/s1.
